# Energy landscape analysis of health checkup data clarified multiple pathways to diabetes development in obese and non-obese subjects

**DOI:** 10.3389/fendo.2025.1576431

**Published:** 2025-05-06

**Authors:** Ryo Ito, Makito Oku, Iwao Kimura, Takayuki Haruki, Masataka Shikata, Tsuyoshi Teramoto, Daisuke Chujo, Minoru Iwata, Shiho Fujisaka, Yoshiki Nagata, Takashi Yamagami, Makoto Kadowaki, Kazuyuki Tobe, Shigeru Saito, Keiichi Ueda

**Affiliations:** ^1^ Graduate School of Science and Engineering, University of Toyama, Toyama, Japan; ^2^ Research Center for Pre-Disease Science, University of Toyama, Toyama, Japan; ^3^ Faculty of Science, University of Toyama, Toyama, Japan; ^4^ Faculty of Sustainable Design, University of Toyama, Toyama, Japan; ^5^ First Department of Internal Medicine, University of Toyama, Toyama, Japan; ^6^ Center for Clinical and Translational Research, Toyama University Hospital, Toyama, Japan; ^7^ Second Department of Human Science, Faculty of Medicine, University of Toyama, Toyama, Japan; ^8^ Laboratory of Preventive Medicine, Hokuriku Health Service Association, Toyama, Japan

**Keywords:** energy landscape analysis, multiple pathways, pre-disease state, specific health checkup data, diabetes, obesity

## Abstract

**Aims:**

To clarify the pathways from a healthy state to the diabetes onset via pre-disease states, we applied energy landscape analysis (ELA) to Specific Health Checkup data in Japan.

**Methods:**

This retrospective and observational cohort study analyzed data from 4,928 males aged 56.0 ± 3.2 years, including 242 individuals with diabetes, over a period of 5.26 ± 3.21 years. A total of 22,326 records were examined using six features: hemoglobin A1c, plasma glucose, high-density lipoprotein-cholesterol, body mass index (BMI), uric acid, and alanine aminotransferase. ELA was also applied to subdata from the 242 individuals with diabetes.

**Results:**

ELA revealed three stable states: healthy, intermediate, and unhealthy (pre-diabetes) states. The intermediate state was characterized by obesity. Obese individuals with BMI ≥ 25 kg/m^2^ (n = 1,460) preferred a pathway via the intermediate state, whereas non-obese individuals with BMI < 25 kg/m^2^ (n = 3,468) preferred to transit directly to the unhealthy state. There was a significant difference between the preferences of the two groups (p = 0.0085, chi-squared test). Two distinct pathways were also observed for obese and non-obese individuals with diabetes.

**Conclusions:**

We demonstrated that ELA could indicate different pathways of diabetes development in obese and non-obese individuals in a data-driven manner. These insights could inform more targeted diabetes prevention measures, such as reducing visceral fat in obese individuals and protecting beta-cells in non-obese individuals.

## 1 Introduction

The number of individuals with diabetes is on the rise around the world (1, 2), and the increasing medical expenses associated with it have become a significant concern. Diabetes can be prevented by implementing appropriate interventions before its onset (3–5). A state between a healthy state and a disease state is generally called a pre-disease state (6–8), and the pre-disease state of diabetes includes pre-diabetes and earlier stages broadly. Early detection of individuals in the pre-disease state having a high risk of diabetes and providing suitable interventions are expected to help control the increase in the number of individuals with diabetes.

The Industrial Safety and Health Act in Japan mandates regular health checkups at least once a year (9). Japanese-style regular health checkups, also known as “Specific Health Checkups,” are basically targeted at all employees aged 40 or over of all companies, and do not focus on high-risk groups for any particular disease. Therefore, oral glucose tolerance test and insulin measurement are not performed due to cost-benefit tradeoffs. On the other hand, an important advantage of Specific Health Checkups is that they allow the longitudinal measurement of many features, such as body mass index (BMI) and plasma glucose (PG) levels, for a large number of individuals. Thus, the time-series data from these health checkups is expected to be useful for early detection of individuals at high risk of diabetes.

Machine learning (ML)-based approaches have been proposed for predicting diabetes (10–19). However, predicting diabetes alone is not enough for effective interventions. It is also necessary to clarify the detailed pathways before the diabetes onset. Currently, methods for extracting such features from health checkup data are not well-established.

Energy landscape analysis (ELA) has been proposed as a technique for understanding state transitions in multidimensional time-series data, and it has been applied to various datasets (20–26). ELA identifies multiple stable states corresponding to energy valleys in the data and represents the difficulty of transitions between states as the height of the energy barrier. Therefore, ELA was expected to be suitable for revealing the important states and pathways before the diabetes onset. Each state is composed of multiple microstates, which are referred to as “patterns” henceforth. Each pattern is assigned a virtual energy level; the lower the energy, the more frequently the pattern will occur. The minimum energy pattern (also called the local minimum pattern) for each state is the most frequent and representative pattern for that state.

As a metaphor, the energy landscape is often explained as a ball on an uneven terrain. Without noise, the ball falls down a slope and reaches the bottom of a valley. With noise, the ball can climb a slope, and there is a small probability that the ball escapes from the current valley and moves to another one. The vertical axis of the terrain corresponds to the virtual energy. However, it should be noted that the “terrain” considered in ELA is not continuous but consists of discrete patterns.

What is unique about ELA is that the value of each feature is converted to 0 or 1 depending on whether the original value is below or above a threshold. Therefore, each pattern corresponds to a sequence of 0 and 1, such as “010111”, meaning the first feature is low, the second feature is high, and so on. This binarization process is believed to help ELA capture the global structure of the data.

In the present study, we applied ELA to Specific Health Checkup data in Toyama Prefecture in Japan to clarify the pathways from a healthy state to the diabetes onset via pre-disease states.

## 2 Materials and methods

### 2.1 Definition of diabetes and obesity

In the present study, an individual was diagnosed with diabetes based on the health checkup data if the individual fulfilled any of the following four conditions according to the diagnostic criteria of the Japanese Clinical Practice Guideline for Diabetes (27): (a) fasting PG ≥ 126 mg/dL and hemoglobin A1c (HbA1c) ≥ 6.5% (48 mmol/mol), (b) non-fasting PG ≥ 200 mg/dL and HbA1c ≥ 6.5% (48 mmol/mol), (c) responded to the questionnaire to have a history of diabetes, and (d) responded to the questionnaire to be under diabetes treatment. Since the questionnaire was administered at each health checkup, some individuals began to respond that they had a history of diabetes or were under diabetes treatment from a certain year. The first year when an individual was diagnosed with diabetes was taken as the diabetes onset. Although the health checkup data did not include information to distinguish between type 1 and type 2 diabetes, such as C-peptide levels, it was expected that most cases of diabetes in the present study were type 2 diabetes based on the general prevalence.

The obesity group was defined as individuals with a BMI of 25 kg/m^2^ or greater at the first measurement according to the diagnostic criteria of the Japan Society for the Study of Obesity (28–30). Similarly, the non-obesity group was defined as individuals with a BMI of less than 25 kg/m^2^ at the first measurement.

### 2.2 Study participants

The participants in the present study were 18,373 individuals aged 55.8 ± 3.3 years (49 to 64 years) who underwent Specific Health Checkups from April 2012 to March 2021 at Hokuriku Health Service Association in Toyama Prefecture in Japan. The anonymized dataset consisted of 108,920 records and 206 features. Among the 18,373 individuals, 1,192 individuals developed diabetes by 2020. The background characteristics of the study participants are shown in **Supplementary Table S1**. A list of abbreviations is shown in **Supplementary Table S2**.

### 2.3 Research design

**Figure 1:**
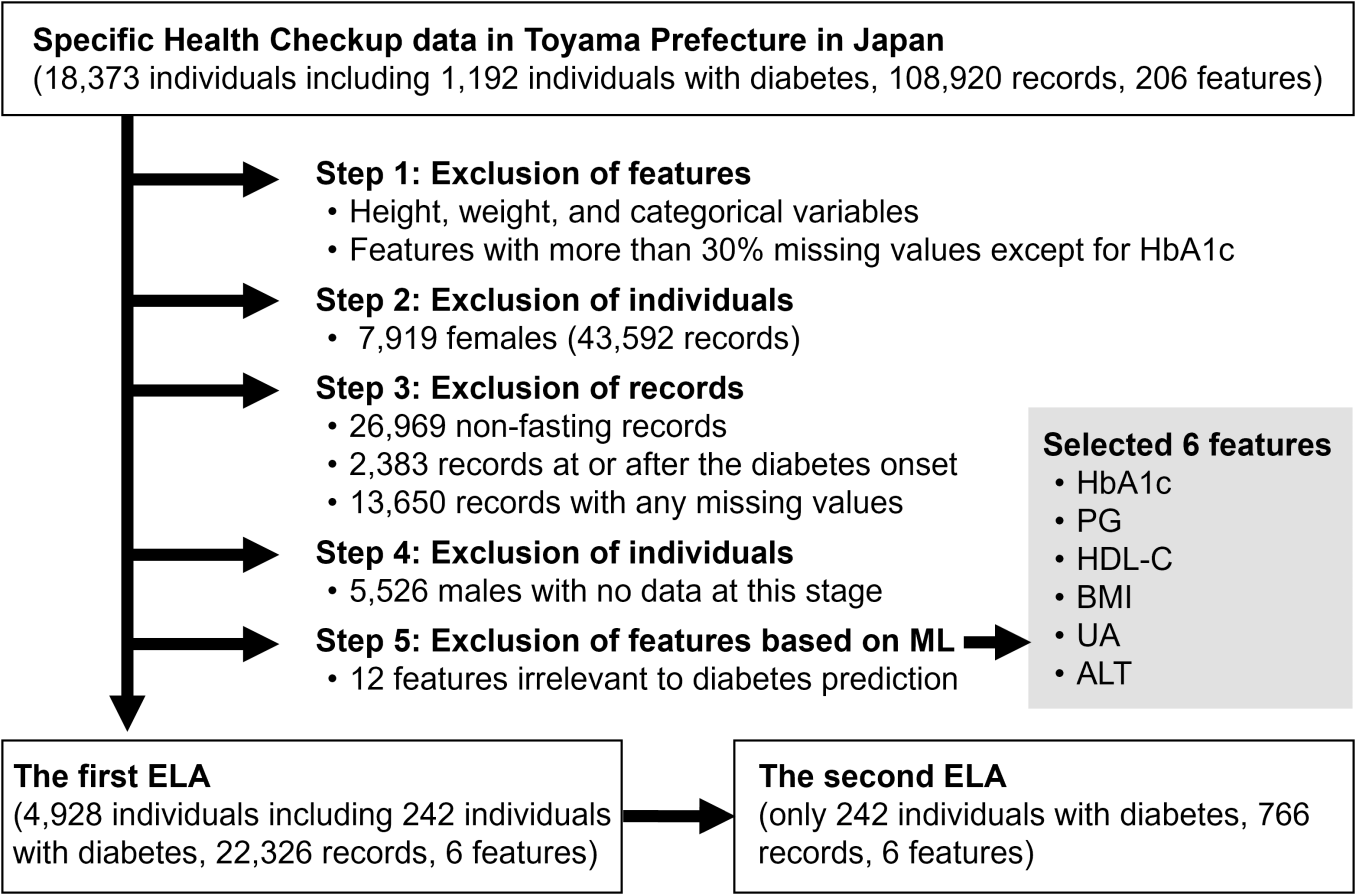
Flowchart of the study design. HbA1c, hemoglobin A1c; PG, plasma glucose; HDL-C, high-density lipoprotein-cholesterol; BMI, body mass index; UA, uric acid; ALT, alanine aminotransferase; ML, machine learning; ELA, energy landscape analysis.

**Figure 1** shows a flowchart of our study design. Before performing ELA, we excluded features, individuals, and records in five steps. In the first step, we excluded redundant or unsuitable features for ELA: height, weight, categorical variables, and features with more than 30% missing values, except for HbA1c. Although HbA1c had 37% missing values, we included it as a clinically important feature because HbA1c generally reflects long-term blood glucose levels and plays a complementary role to PG (31). 18 features remained at this step: BMI, waist circumference (WC), systolic blood pressure (SBP), diastolic blood pressure (DBP), triglycerides (TG), low-density lipoprotein-cholesterol (LDL-C), high-density lipoprotein-cholesterol (HDL-C), PG, HbA1c, uric acid (UA), creatinine (Cre), aspartate aminotransferase (AST), alanine aminotransferase (ALT), γ-glutamyl transpeptidase (γ-GTP), white blood cell count (WBC), red blood cell count (RBC), hemoglobin (Hb), and hematocrit (Ht).

In the second step, we excluded 7,919 females and their corresponding 43,592 records. We considered the influence of estrogen on insulin resistance and conducted the analysis exclusively on males. In the third step, we excluded records that met any of the following criteria: (a) records with non-fasting blood sampling (26,969 records), (b) records at or after the diabetes onset (2,383 records), and (c) records with any missing values among the 18 features (13,650 records). In the fourth step, we excluded 5,526 males with no data at this stage.

In the fifth step, we excluded features again based on ML prediction analysis. A random forest model was trained to predict whether an individual would develop diabetes within three years when given a single-year record of the individual on the 17 features except for HbA1c. The top five features that contributed most to predicting diabetes onset were PG, HDL-C, BMI, UA, and ALT. We added HbA1c to them as a clinically important feature, and the six features were selected for the following ELA. The 12 other features were excluded.

Finally, a subset of 22,326 records and 6 features from 4,928 individuals (males only, aged 56.0 ± 3.2 years) over a period of 5.26 ± 3.21 years was obtained and was used for the first ELA. Among the 4,928 individuals, 242 developed diabetes by 2020. The second ELA used the same data as the first ELA but was limited to 766 records from the 242 individuals with diabetes.

Our previous study (Shikata et al., unpublished) analyzed the same dataset from a completely different perspective. It focused on weight changes before diabetes onset and did not use ELA, whereas the present study used ELA to characterize multivariate transitions before diabetes onset. Therefore, the present study is complementary to the previous study.

### 2.4 Energy landscape analysis

The basic steps of ELA are feature selection, data binarization, Ising model fitting, basin graph calculation, and disconnectivity graph calculation. Detailed mathematical explanations are provided in **Supplementary Text**. In the feature selection step, several features to be analyzed should be selected. Using a large number of features not only increases the computational cost, but can lead to unreliable results. In the data binarization step, the values of the specified six features were converted to 0 or 1. To represent good health conditions as 0 and bad health conditions as 1, values less than or equal to the median of each feature were converted to 0, and all other values were converted to 1, except for HDL-C. For HDL-C, the assignment was reversed. After the binarization, the entire dataset was categorized into 2^6^ = 64 patterns.

In the calculation of a basin graph, each pattern was expressed in a decimal number. For example, a pattern of “010111” was transformed to 0 + 16 + 0 + 4 + 2 + 1 = 23. For each pattern or node, a directed edge was drawn toward the node with the lowest energy among its neighborhood except for the local minimum patterns. We regarded each set of connected nodes of the basin graph as a “state.”

In the calculation of a disconnectivity graph, the height of the energy barrier for each pair of states was calculated. We also calculated a modified disconnectivity graph in which visits to patterns outside the two target states were prohibited.

We also counted the occurrence of state transitions. Two consecutive years of measurements of the same individual were analyzed. For example, when an individual was in state 1 in 2012 and changed to state 2 in 2013, the number of transitions from state 1 to state 2 was counted as 1. Transitions to the same state were included as self-transitions. Transitions between measurements separated by two years or more due to missing data were not counted.

### 2.5 Other statistical analysis

Mann-Whitney’s U-test and chi-squared test were used for between-group comparisons on numerical and categorical variables, respectively. Numerical variables were presented as the mean ± standard deviation. A p-value less than 0.05 was considered statistically significant.

### 2.6 Software

All statistical analyses were performed with Python version 3.10 (Python Software Foundation, Beaverton, OR, USA). We ported the original MATLAB code of ELA (32) to Python and used it.

### 2.7 Ethical approval

All procedures of the present study were performed in accordance with the 1964 Helsinki Declaration, its later amendments, and the “Ethical Guidelines for Medical and Biological Research Involving Human Subjects” published by the Ministry of Health, Labour and Welfare of Japan. The ethics committee of Toyama University Hospital approved the study protocol (approval number: R2021070, approved date: 2021/8/19). The study participants consented to the use of their data for scientific research.

### 2.8 Data and resource availability

The datasets generated during and/or analyzed in the current study are available from the corresponding authors upon reasonable request. The Python port of the ELA toolbox used in the current study is available in a GitHub repository https://github.com/okumakito/elapy under the Apache 2.0 license.

## 3 Results

### 3.1 Results of the first ELA

**Figure 2:**
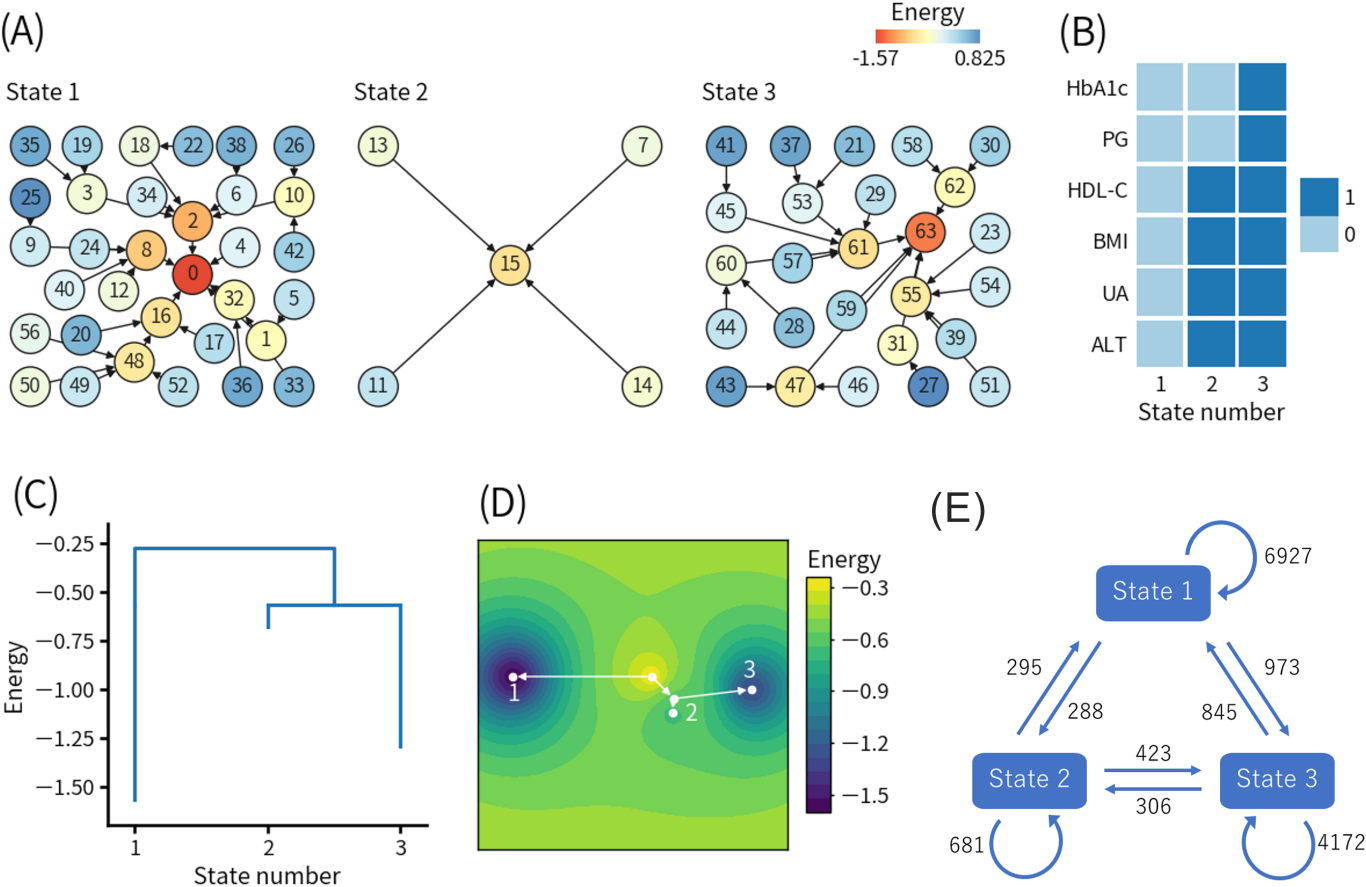
Results of the first energy landscape analysis. **(A)** Basin graph. The node number indicates the decimal representation of each binary pattern (see detail in the main text). The node color indicates the energy level of the node. Edges are drawn toward the minimum energy node within each node’s neighborhood. **(B)** Local minimum patterns of each state. 0 and 1 indicate relatively good and bad health conditions, respectively. **(C)** Disconnectivity graph. The height of paths connecting states indicates the height of energy barriers between states. The energy value for each state indicates the energy of the local minimum pattern of the state. **(D)** Energy landscape reconstructed from the disconnectivity graph. Movement is only allowed on the white lines. **(E)** State transition counts. The numbers in the figure indicate the total numbers of the events. HbA1c, hemoglobin A1c; PG, plasma glucose; HDL-C, high-density lipoprotein-cholesterol; BMI, body mass index; UA, uric acid; ALT, alanine aminotransferase.

**Figure 2** shows the results of the first ELA. The threshold values for the binarization were as follows: HbA1c, 5.6% (38 mmol/mol); PG, 95 mg/dL; HDL-C, 58 mg/dL; BMI, 23.3 kg/m^2^; UA, 6.1 mg/dL; and ALT, 22 U/L. **Figure 2A** shows the basin graph, which was separated into three states. The numbers of patterns belonging to states 1, 2, and 3 were 33, 5, and 26, respectively. The local minimum patterns of states 1, 2, and 3 were numbered 0, 15, and 63, respectively. The energy level of pattern 15 was higher than pattern 0 and pattern 63, indicating that state 2 was less frequent. Pattern 0 had the lowest energy level, indicating that state 1 was the most frequent.

**Figure 2B** shows the local minimum patterns of the three states. As explained earlier, the binary representations 0 and 1 indicate relatively good and bad health conditions, respectively. Therefore, states 1, 2, and 3 corresponded to healthy, intermediate, and unhealthy states in a relative sense, respectively.

**Figure 2C** shows the disconnectivity graph, and **Figure 2D** shows the energy landscape reconstructed from the disconnectivity graph. The energy barrier for the transition from state 1 to state 2 or 3 was higher than the energy barrier between state 2 and state 3, which was also close to the minimum energy level of state 2. Namely, the transition from state 1 to state 2 or 3 was more difficult than the transition between state 2 and state 3.

In addition, the modified disconnectivity graph shown in **Supplementary Figure S1A** revealed that the energy barrier between state 1 and state 3 increased when visits to state 2 were prohibited. Therefore, the direct transition from state 1 to state 3 (1-3 pathway) required to pass through a pattern with higher energy (i.e., a lower frequency of occurrence) than the transition from state 1 to state 3 via state 2 (1-2-3 pathway).

**Figure 2E** shows the state transition counts. The numbers of self-transitions of states 1, 2, and 3 were 6927, 681, and 4172, respectively, which were consistent with the numbers of patterns belonging to each state, as shown in **Figure 2A**. The numbers of transitions from state 1 to state 2 and from state 2 to state 3, which corresponded to the 1-2-3 pathway, were 288 and 423, respectively. Interestingly, the number of transitions directly from state 1 to state 3 was 973, which was more than twice the occurrences of the 1-2-3 pathway. Focusing on state 2, the number of transitions from state 2 to state 3 was higher than transitions from state 2 to state 1, which was also consistent with the disconnectivity graph, as shown in **Figure 2C**.

To identify the features contributing to the choice between the 1-2-3 pathway and the 1-3 pathway, we compared the values of each feature in the year before the transition for each type of state transition, as shown in **Table 1**. In the comparison between the transition from state 1 to state 2 and the transition from state 1 to state 3, significant differences were observed in BMI (24.0 ± 2.3 kg/m^2^ vs 23.3 ± 2.3 kg/m^2^, p ≤ 0.0001), WC (85.9 ± 6.8 cm vs 84.6 ± 6.7 cm, p ≤ 0.05), HDL-C (57.0 ± 10.9 mg/dL vs 60.5 ± 12.9 mg/dL, p ≤ 0.001), PG (88.5 ± 6.3 mg/dL vs 97.0 ± 9.7 mg/dL, p ≤ 0.0001), HbA1c (5.37 ± 0.19% (35.2 ± 2.1 mmol/mol) vs 5.69 ± 0.29% (38.7 ± 3.2 mmol/mol), p ≤ 0.0001), Cre (0.87 ± 0.13 mg/dL vs 0.86 ± 0.25 mg/dL, p ≤ 0.05), ALT (23.2 ± 11.1 U/L vs 21.2 ± 9.2 U/L, p ≤ 0.001), Hb (15.3 ± 1.0 g/dL vs 15.0 ± 1.0 g/dL, p ≤ 0.0001), and Ht (45.3 ± 2.6% vs 44.7 ± 2.8%, p ≤ 0.01).

**Table 1:** Comparison of feature values in the year before the transition for the first ELA†.

Feature	state 1 to 2 (healthy to intermediate state)	state 1 to 3 (healthy to unhealthy state, direct)	state 2 to 1 (intermediate to healthy state, recovering)	state 2 to 3 (intermediate to unhealthy state)	p-value (state 1 to 2 vs state 1 to 3)	p-value (state 1 to 3 vs state 2 to 3)	p-value (state 2 to 1 vs state 2 to 3)
BMI (kg/m^2^)	24.0 ± 2.3	23.3 ± 2.3	24.2 ± 2.3	25.8 ± 2.7	≤ 0.0001	≤ 0.0001	≤ 0.0001
WC (cm)	85.9 ± 6.8	84.6 ± 6.7	86.6 ± 6.6	90.2 ± 7.6	≤ 0.05	≤ 0.0001	≤ 0.0001
SBP (mmHg)	126.1 ± 14.6	127.1 ± 15.0	126.8 ± 13.6	129.2 ± 14.9	0.44	≤ 0.01	≤ 0.05
DBP (mmHg)	79.9 ± 11.0	79.8 ± 10.6	80.8 ± 10.6	81.6 ± 11.3	0.69	≤ 0.01	0.42
TG (mg/dL)	131.9 ± 103.0	122.1 ± 83.7	171.8 ± 222.0	162.6 ± 111.3	0.06	≤ 0.0001	0.10
LDL-C (mg/dL)	130.1 ± 27.1	129.9 ± 30.3	128.4 ± 33.1	132.0 ± 32.1	0.75	0.21	0.12
HDL-C (mg/dL)	57.0 ± 10.9	60.5 ± 12.9	53.1 ± 10.1	50.9 ± 11.3	≤ 0.001	≤ 0.0001	≤ 0.0001
PG (mg/dL)	88.5 ± 6.3	97.0 ± 9.7	88.2 ± 4.9	90.1 ± 4.4	≤ 0.0001	≤ 0.0001	≤ 0.0001
HbA1c (%)	5.37 ± 0.19	5.69 ± 0.29	5.37 ± 0.2	5.47 ± 0.16	≤ 0.0001	≤ 0.0001	≤ 0.0001
HbA1c (mmol/mol)	35.2 ± 2.1	38.7 ± 3.2	35.2 ± 2.2	36.3 ± 1.7	≤ 0.0001	≤ 0.0001	≤ 0.0001
UA (mg/dL)	6.16 ± 1.07	6.02 ± 1.2	6.72 ± 1.03	6.81 ± 1.11	0.055	≤ 0.0001	0.39
Cre (mg/dL)	0.87 ± 0.13	0.86 ± 0.25	0.88 ± 0.13	0.90 ± 0.15	≤ 0.05	≤ 0.0001	≤ 0.05
AST (U/L)	24.3 ± 15.6	22.7 ± 7.6	26.1 ± 10.7	26.8 ± 9.7	0.49	≤ 0.0001	0.12
ALT (U/L)	23.2 ± 11.1	21.2 ± 9.2	27.8 ± 11.0	32.8 ± 16.0	≤ 0.001	≤ 0.0001	≤ 0.0001
γ-GTP (U/L)	55.6 ± 78.5	51.5 ± 52.6	65.1 ± 85.6	62.6 ± 53.7	0.15	≤ 0.0001	0.10
WBC (1/μL)	6186 ± 1737	6096 ± 1632	6191 ± 1744	6225 ± 1552	0.52	0.07	0.43
RBC (10k/μL)	489.0 ± 34.6	484.6 ± 37.5	493.9 ± 35.6	495.8 ± 37.8	0.11	≤ 0.0001	0.39
Hb (g/dL)	15.3 ± 1.0	15.0 ± 1.0	15.4 ± 1.0	15.4 ± 1.0	≤ 0.0001	≤ 0.0001	0.97
Ht (%)	45.3 ± 2.6	44.7 ± 2.8	45.6 ± 2.9	45.5 ± 2.7	≤ 0.01	≤ 0.0001	0.97

^†^Numerical variables were presented as the mean ± standard deviation. BMI, Body mass index; WC, Waist circumference; SBP, Systolic blood pressure; DBP, Diastolic blood pressure; TG, Triglyceride; LDL-C, Low-density lipoprotein-cholesterol, HDL-C, High-density lipoprotein-cholesterol; PG, Plasma glucose; HbA1c, Hemoglobin A1c/Glycated hemoglobin; UA, Uric acid; Cre, Creatinine; AST, Aspartate aminotransferase; ALT, Alanine aminotransferase; γ-GTP, γ-glutamyl transpeptidase, WBC, White blood cell count; RBC, Red blood cell count; Hb, Hemoglobin; Ht, Hematocrit; ELA, energy landscape analysis.

In the comparison between the transition from state 1 to state 3 and the transition from state 2 to state 3 (**Table 1**), significant differences were observed in BMI (23.3 ± 2.3 kg/m^2^ vs 25.8 ± 2.7 kg/m^2^, p ≤ 0.0001), WC (84.6 ± 6.7 cm vs 90.2 ± 7.6 cm, p ≤ 0.0001), SBP (127.1 ± 15.0 mmHg vs 129.2 ± 14.9 mmHg, p ≤ 0.01), DBP (79.8 ± 10.6 mmHg vs 81.6 ± 11.3 mmHg, p ≤ 0.01), TG (122.1 ± 83.7 mg/dL vs 162.6 ± 111.3 mg/dL, p ≤ 0.0001), HDL-C (60.5 ± 12.9 mg/dL vs 50.9 ± 11.3 mg/dL, p ≤ 0.0001), PG (97.0 ± 9.7 mg/dL vs 90.1 ± 4.4 mg/dL, p ≤ 0.0001), HbA1c (5.69 ± 0.29% (38.7 ± 3.2 mmol/mol) vs 5.47 ± 0.16% (36.3 ± 1.7 mmol/mol), p ≤ 0.0001), UA (6.02 ± 1.2 mg/dL vs 6.81 ± 1.11 mg/dL, p ≤ 0.0001), Cre (0.86 ± 0.25 mg/dL vs 0.90 ± 0.15 mg/dL, p ≤ 0.0001), AST (22.7 ± 7.6 U/L vs 26.8 ± 9.7 U/L, p ≤ 0.0001), ALT (21.2 ± 9.2 U/L vs 32.8 ± 16.0 U/L, p ≤ 0.0001), γ-GTP (51.5 ± 52.6 U/L vs 62.6 ± 53.7 U/L, p ≤ 0.0001), RBC (484.6 ± 37.5 × 10^4^/μL vs 495.8 ± 37.8 × 10^4^/μL, p ≤ 0.0001), Hb (15.0 ± 1.0 g/dL vs 15.4 ± 1.0 g/dL, p ≤ 0.0001), and Ht (44.7 ± 2.8% vs 45.5 ± 2.7%, p ≤ 0.0001). Interestingly, PG and HbA1c levels were significantly higher in the transition from state 1 to 3 (1-3 pathway) than the transition from state 2 to 3 (1-2-3 pathway).

In addition, we compared the transition from state 2 to state 1 and the transition from state 2 to state 3 to identify features contributing to whether individuals in state 2 return to state 1 or worsen to state 3, as shown in **Table 1**. In that comparison, significant differences were observed in BMI (24.2 ± 2.3 kg/m^2^ vs 25.8 ± 2.7 kg/m^2^, p ≤ 0.0001), WC (86.6 ± 6.6 cm vs 90.2 ± 7.6 cm, p ≤ 0.0001), SBP (126.8 ± 13.6 mmHg vs 129.2 ± 14.9 mmHg, p ≤ 0.05), HDL-C (53.1 ± 10.1 mg/dL vs 50.9 ± 11.3 mg/dL, p ≤ 0.0001), PG (88.2 ± 4.9 mg/dL vs 90.1 ± 4.4 mg/dL, p ≤ 0.0001), HbA1c (5.37 ± 0.2% (35.2 ± 2.2 mmol/mol) vs 5.47 ± 0.16% (36.3 ± 1.7 mmol/mol), p ≤ 0.0001), Cre (0.88 ± 0.13 mg/dL vs 0.90 ± 0.15 mg/dL, p ≤ 0.05), and ALT (27.8 ± 11.0 U/L vs 32.8 ± 16.0 U/L, p ≤ 0.0001).

Because BMI was identified as one of the main factors determining the direction of state transitions, we stratified the individuals into the obese group with BMI ≥ 25 kg/m^2^ (n = 1,460) and the non-obese group with BMI < 25 kg/m^2^ (n = 3,468). **Supplementary Figure S2** shows the state transition counts for the obese and non-obese groups. For the obese group, the numbers of transitions from state 1 to state 2, state 2 to state 3, and state 1 to state 3 were 78, 222, and 191, respectively. For the non-obese group, the numbers of transitions from state 1 to state 2, state 2 to state 3, and state 1 to state 3 were 210, 201, and 782, respectively. The obese group significantly preferred the transition from state 1 to state 2 over the transition from state 1 to state 3 than the non-obese group (p = 0.0085). The obese group also significantly preferred the transition from state 2 to state 3 over the transition from state 1 to state 3 than the non-obese group (p < 0.0001).

### 3.2 Results of the second ELA limited to individuals with diabetes

**Figure 3:**
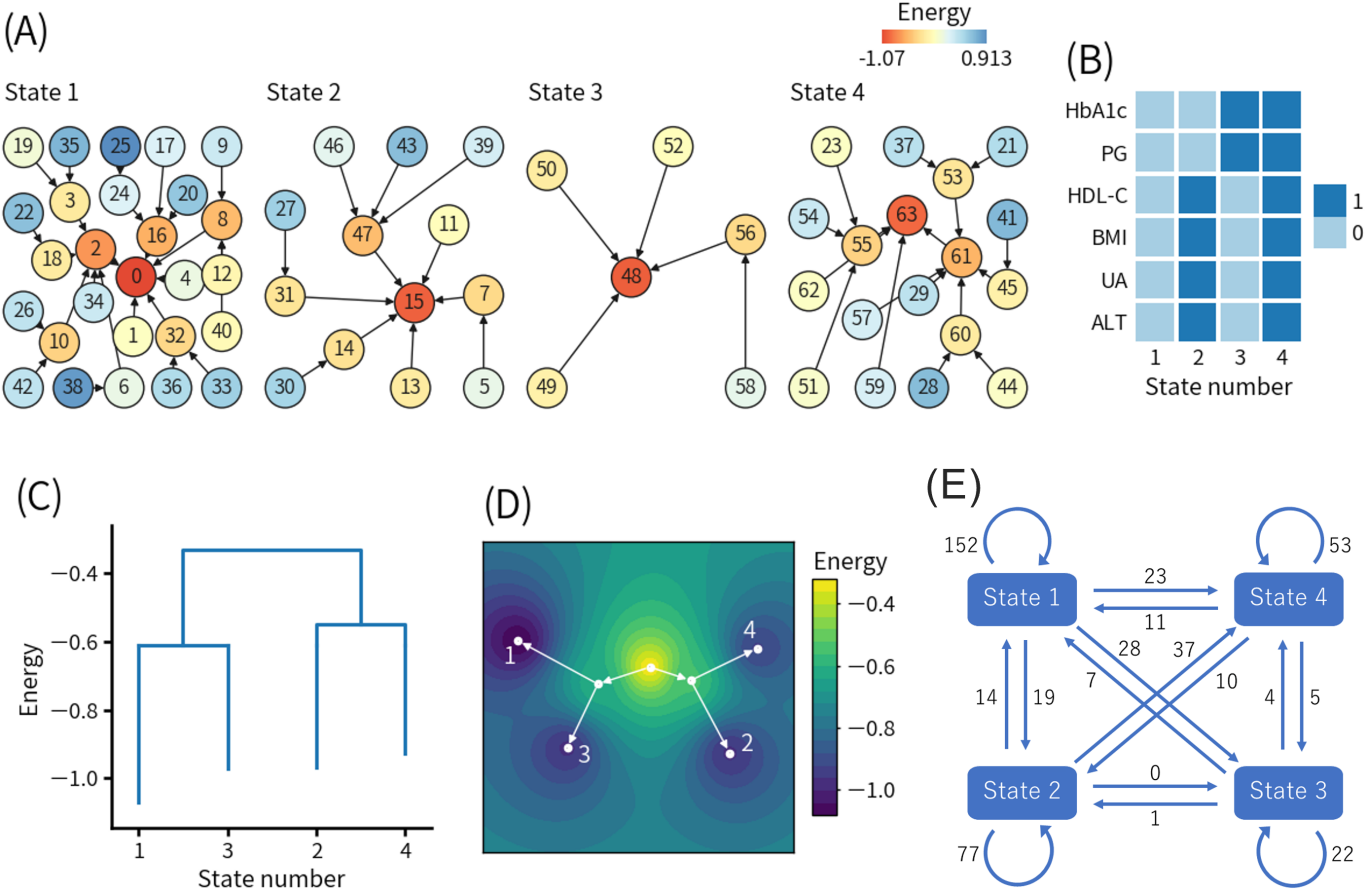
Results of the second energy landscape analysis. **(A)** Basin graph. The node number indicates the decimal representation of each binary pattern (see detail in the main text). The node color indicates the energy level of the node. Edges are drawn toward the minimum energy node within each node’s neighborhood. **(B)** Local minimum patterns of each state. 0 and 1 indicate relatively good and bad health conditions, respectively. **(C)** Disconnectivity graph. The height of paths connecting states indicates the height of energy barriers between states. The energy value for each state indicates the energy of the local minimum pattern of the state. **(D)** Energy landscape reconstructed from the disconnectivity graph. Movement is only allowed on the white lines. **(E)** State transition counts. The numbers in the figure indicate the total numbers of the events. HbA1c, hemoglobin A1c; PG, plasma glucose; HDL-C, high-density lipoprotein-cholesterol; BMI, body mass index; UA, uric acid; ALT, alanine aminotransferase.

The second ELA targeted only the data of the 242 individuals who developed diabetes by 2020. As with the first ELA, all records at or after the diabetes onset were excluded to focus on the period before diabetes onset. **Figure 3** shows the results of the second ELA. The threshold values for the data binarization were as follows: HbA1c, 6.2% (44 mmol/mol); PG, 112 mg/dL; HDL-C, 49 mg/dL; BMI, 25.8 kg/m^2^; UA, 6.1 mg/dL; and ALT, 33 U/L. It should be noted that the meanings of patterns with the same numbers in **Figures 2**, **3** are quantitatively different.

**Figure 3A** shows the basin graph, which was separated into four states. The numbers of patterns belonging to states 1, 2, 3, and 4 were 27, 13, 6, and 18, respectively. The local minimum patterns of states 1, 2, 3, and 4 were numbered 0, 15, 48, and 63, respectively.

**Figure 3B** shows the local minimum patterns of the four states. State 1 and state 4 corresponded to healthy and unhealthy states in a relative sense. State 2 and state 3 seemingly corresponded to different types of intermediate states between state 1 and state 4. However, state 3 had high HbA1c and PG levels, and there were not many transitions from state 3 to state 4, indicating that there were direct transitions from state 3 to diabetes. Only state 3 was not found in the first ELA.

**Figure 3C** shows the disconnectivity graph, and **Figure 3D** shows the energy landscape reconstructed from the disconnectivity graph. The energy barriers between state 1 and state 3 and between state 2 and state 4 were much lower than the energy barrier for the transition from state 1 or 3 to state 2 or 4. In other words, state 1 was more likely to transition to state 3, and state 2 was more likely to transition to state 4 compared to other states. In both the transition from state 1 to state 3 (1-3 pathway) and the transition from state 2 to state 4 (2-4 pathway), HbA1c and PG increased and exceeded the binarization thresholds, whereas the other four features did not change beyond the binarization thresholds. The modified disconnectivity graph shown in **Supplementary Figure S1B** revealed that the energy barrier between state 1 and state 4 increased when visits to state 2 and state 3 were prohibited.

**Figure 3E** shows the state transition counts. The self-transitions of states 1, 2, 3, and 4 were 152, 77, 22, and 53 times, respectively. Except for the self-transitions, the top two most frequent transitions were 37 times from state 2 to state 4 and 28 times from state 1 to state 3, which were consistent with the disconnectivity graph, as shown in **Figure 3C**.

To clarify the characteristics of the 1-3 pathway and the 2-4 pathway, we compared the transition from state 1 to state 3 and the transition from state 2 to state 4, as shown in **Supplementary Table S3**. In that comparison, significant differences were observed in BMI (23.8 ± 2.3 kg/m^2^ vs 29.3 ± 3.4 kg/m^2^, p ≤ 0.0001), WC (84.9 ± 5.0 cm vs 99.1 ± 8.9 cm, p ≤ 0.0001), DBP (78.7 ± 10.1 mmHg vs 84.5 ± 9.7 mmHg, p ≤ 0.01), TG (126.7 ± 64.0 mg/dL vs 181.6 ± 91.4 mg/dL, p ≤ 0.01), HDL-C (55.3 ± 13.0 mg/dL vs 44.6 ± 9.0 mg/dL, p ≤ 0.001), PG (112.0 ± 6.6 mg/dL vs 108.6 ± 7.3 mg/dL, p ≤ 0.05), UA (5.82 ± 1.36 mg/dL vs 7.22 ± 1.22 mg/dL, p ≤ 0.001), AST (23.9 ± 7.5 U/L vs 37.2 ± 16.0 U/L, p ≤ 0.0001), ALT (26.7 ± 15.9 U/L vs 53.0 ± 23.9 U/L, p ≤ 0.0001), and γ-GTP (62.0 ± 92.5 U/L vs 76.6 ± 60.9 U/L, p ≤ 0.01). Interestingly, PG level was significantly higher in the transition from state 1 to 3 than the transition from state 2 to 4.

## 4 Discussion

### 4.1 Summary of key findings

The present study demonstrated that ELA could indicate different pathways of diabetes development in obese and non-obese individuals in a data-driven manner. Previous studies reported that type 2 diabetes can be divided into five subgroups using machine learning methods (33–36). It was also reported that type 2 diabetes can be classified into eight subgroups using genome-wide association studies (37). Our findings suggest that the recently emerging idea that diabetes is more heterogeneous than previously thought applies even before onset. Specifically, we found that obese individuals significantly preferred to visit an intermediate state with increased BMI and deteriorated HDL-C, UA, and ALT levels. We also found that non-obese individuals were at risk for elevated PG and HbA1c without passing through the intermediate state. These insights could inform more targeted diabetes prevention measures, such as reducing visceral fat in obese individuals and protecting beta-cells in non-obese individuals. Detailed discussion is given below.

### 4.2 Comparison of ELA and other methods

Our findings suggest that ELA is a powerful tool for investigating trajectories before the diabetes onset. A previous study used a linear mixed model to analyze trajectories of BMI and WC before the diabetes onset (38). It reported that individuals who progressed from pre-diabetes to diabetes showed significantly greater pre-onset increases in both BMI and WC compared to individuals who remained in pre-diabetes. Another previous study used a latent class trajectory analysis, in which a linear mixed model was used, to analyze BMI trajectories before the diabetes onset (39). It reported that even in the normoglycemic group, an increasing BMI trend was associated with the risk of type 2 diabetes. In contrast to these linear models, ELA can capture nonlinear trajectories, such as multiple transitions between a healthy state and a pre-disease state, i.e., repeated declines and recoveries in health status. Therefore, ELA is expected to work as a complementary tool to existing linear models and contribute to adding new insights.

Cluster analysis is another powerful method. Using it, previous studies successfully identified novel subgroups of diabetes in a data-driven manner (33, 34). Although there have been no studies comparing cluster analysis and ELA in detail, cluster analysis may be able to detect multiple stable states before the diabetes onset, similar to ELA. However, it is generally believed that ELA is suitable for cases where the status of the target system (for example, an individual’s health status) switches between multiple states over time (20–26). Another difference is that the data binarization in ELA may improve noise tolerance at the expense of loss of quantitative information. It is an important future work to clarify the advantages and disadvantages of ELA against cluster analysis as well as other existing methods.

### 4.3 Interpretation of the pre-disease states identified in the first ELA

The results of the first ELA suggest that multiple pre-disease states exist between the healthy state and the diabetes onset. It should be noted that the unhealthy state does not correspond to diabetes because we removed all records at or after the diabetes onset from the analysis. We expect that the unhealthy state mainly corresponds to pre-diabetes, which is impaired fasting glucose (IFG) and/or impaired glucose tolerance (IGT). Pre-diabetes is a well-known pre-disease state of diabetes with established diagnostic criteria (27, 40). It is natural that the unhealthy state corresponding to pre-diabetes was detected as a distinct pre-disease state in our analysis.

On the other hand, the intermediate state (another pre-disease state) had fasting PG less than 95 mg/dL and HbA1c less than 5.6% (38 mmol/mol), and thus it is at least not IFG, and probably not IGT either. Previous studies reported that even before pre-diabetes, higher fasting PG was associated with a higher risk of type 2 diabetes (41). The idea that the stages before pre-diabetes are also clinically important is consistent with our results. Previous studies also reported that UA and ALT were risk factors for diabetes (42, 43), which is consistent with our results. Because we detected the intermediate state using a data-driven approach without *a priori* assumptions, our findings suggest the importance of further research on such early stages.

### 4.4 Interpretation of the multiple pathways identified in the first ELA

Next, our results suggest that obese individuals with visceral fat accumulation tended to visit the intermediate state (1-2-3 pathway), whereas non-obese individuals with relatively high PG and HbA1c levels tended to directly transit to the unhealthy state (1-3 pathway). A possible explanation is that the individuals who visited the intermediate state had higher potential capacity for insulin secretion than the others. Insulin secretion generally promotes blood glucose uptake by muscle and adipose tissues (44), lowering PG immediately and HbA1c over a longer time scale. The glucose taken up by the adipose tissues was converted to TG for storage, contributing to the increases in BMI and WC. As abdominal obesity progresses, insulin resistance occurs, and insulin secretion increases due to the compensatory response of beta-cells (45, 46). Taken together, the intermediate state with high BMI and relatively low PG and HbA1c levels may correspond to a state with mild insulin resistance before reaching the maximum capacity of insulin secretion.

In contrast, individuals with insufficient insulin secretion capacity should have relatively high PG and HbA1c, but at the same time, adipose tissue hypertrophy may be suppressed. Non-obese people generally have lower potential insulin secretion capacity than obese people (47). In addition, previous studies have revealed that insulin sensitivity and insulin secretion are inversely related (45, 46).

Therefore, the 1-2-3 and 1-3 pathways may be relatively associated with insulin resistance and beta-cell dysfunction, respectively. This interpretation is consistent with the drug selection algorithm for patients with type 2 diabetes in Japan that basically assumes insulin resistance in obese patients and insulin secretion deficiency in non-obese patients (48). Our study demonstrated in a data-driven manner that the widely recognized differences between obese and non-obese patients with diabetes also apply before the onset.

Previous studies also reported that East Asians including Japanese have lower insulin secretion capacity than Caucasian, and the decline of insulin secretion capacity is a major factor in the development and progression of glucose intolerance in Japanese people even at an early stage (49–54). This may explain why more individuals took the 1-3 pathway than the 1-2-3 pathway.

### 4.5 Interpretation of the results of the second ELA

The results of the second ELA suggest that obese and non-obese individuals tended to take different pathways, which is consistent with the first ELA. The 1-2-3 and 1-3 pathways of the first ELA may primarily correspond to the 2-4 and 1-3 pathways of the second ELA, respectively. An important finding is that non-obese individuals may exhibit increases in HbA1c and PG without remarkable changes in HDL-C, BMI, UA, and ALT. As we argued for the first ELA, such a pathway would be mainly associated with beta-cell dysfunction, which is common in Japanese people.

### 4.6 Clinical implications

In the first ELA, we found that those who could return from the intermediate state to the healthy state had significantly lower BMI and WC than those who deteriorated from the intermediate state to the unhealthy state. This suggests that reducing body weight and visceral fat in the intermediate state through lifestyle guidance and administration of anti-obesity drugs is effective for diabetes prevention, which is consistent with common understanding.

In both the first ELA and second ELA, we found that non-obese individuals preferred to transition directly to a state of elevated HbA1c and PG levels, which corresponds to a pre-disease state of diabetes. Therefore, non-obese individuals should also be careful about their HbA1c and PG levels, and when their HbA1c and PG levels become high, they are recommended to improve their lifestyle habits such as exercise and diet to protect beta-cells and prevent diabetes. It has also been reported that changing the order of meals, such as taking dietary fiber before carbohydrates, effectively suppresses postprandial hyperglycemia (55), which damages beta-cells.

### 4.7 Limitations

The present study has several limitations. First, the present study focused on men aged 55.8 ± 3.3 years who underwent Specific Health Checkups in Toyama Prefecture from April 2012 to March 2021. This selection bias must be considered when interpreting the results. Recent studies have highlighted the importance of diabetes not only for older men but also for women and young people (56, 57). Second, the average observation period was about five years, which is shorter than the typical period from a healthy state without visceral fat accumulation to metabolic syndrome and ultimately diabetes. Our findings should be limited to phenomena relatively close to the diabetes onset. Third, the health checkup data analyzed in the present study did not contain data on insulin, race, family health history, and accurate diagnosis including discrimination between type 1 and type 2 diabetes as well as more detailed stratification (34–37). Fourth, the results of the ELA were based on only six features. This is a general limitation of ELA in that increasing the number of features remarkably reduces the reliability of the results.

It is future tasks to apply ELA to females, younger or elder individuals, data from other regions, long-term data, detailed clinical data, and different combinations of features relevant to other lifestyle diseases. In particular, prospective studies in relatively small populations including oral glucose tolerance test and insulin measurements would be useful to verify the clinical relevance of our findings.

## Data Availability

The data analyzed in this study is subject to the following licenses/restrictions: the participants in this study did not consent to have their data publicly available. Requests to access these datasets should be directed to Makito Oku, oku@cts.u-toyama.ac.jp; Keiichi Ueda, kueda@sci.u-toyama.ac.jp.
