# Supplementary material for: Energy landscape analysis of health checkup data clarified multiple pathways to diabetes development in obese and non-obese subjects
Source: Front Endocrinol (Lausanne). 2025 May 6;16:1576431. doi: 10.3389/fendo.2025.1576431 (PMC12088973; doi:10.3389/fendo.2025.1576431)
Supplement: Supplementary file 1 [file DataSheet1.pdf]

Supplementary Material

1 Supplementary Text

1.1 Mathematical Explanation of Energy Landscape Analysis

Let $x = (x_1, \dots, x_N)^T, \forall x_i \in \{-1,1\}$ be an N-dimensional random variable. An Ising model defines the energy function E(x) using a column vector $h \in \mathbb{R}^N$ and a symmetric matrix $J \in \mathbb{R}^{N \times N}, \forall J_{ii} = 0$ as follows:

$$E(x) = -\frac{1}{2}x^T J x - h^T x. \tag{1}$$

The joint probability distribution P(x) is defined as the Boltzmann distribution using the energy function as follows:

$$P(x) = P(x|h,J) = \frac{1}{Z}\exp\big(-E(x)\big), \tag{2}$$

where Z is the normalization constant that satisfies $\sum_x P(x) = 1$.

Let $y(t) = \big(y_1(t), \dots, y_N(t)\big)^T, \forall y_i \in \{0,1\}, t = 1, \dots, T$ be the t-th observation of the binarized data. By using $x(t) = 2y(t) - 1$, the likelihood function L is defined as follows:

$$L(h,J) = \prod_{t=1}^{T} P(x(t)|h,J). \tag{3}$$

The maximum likelihood estimation of h and J are calculated using an iterative algorithm[1].

$$(h,J) = \underset{h',J'}{\operatorname{argmax}}\, L(h',J'). \tag{4}$$

Briefly, the parameters h and J are updated repeatedly so that the theoretical distribution of the Ising model P(x) approaches the empirical distribution of the data. For more details, see Reference 1.

The disconnectivity graph shows when each state is disconnected (cannot transition to each other) as the energy gradually decreases from its maximum value. In other words, it indicates the height of

the energy barrier between each state. Precisely, the disconnectivity graph and modified disconnectivity graph indicate the energy of the saddle point patterns defined by the following equations, respectively.

$$\tilde{E}_{k\leftrightarrow k'} = \min_{n} \max_{i}\{E_i | i \in p_n, p_n \in P_{k\leftrightarrow k'}\}$$

$$\hat{E}_{k\leftrightarrow k'} = \min_{n} \max_{i}\{E_i | i \in p_n, p_n \in \hat{P}_{k\leftrightarrow k'}\}$$

for k, k' = 1,…K, $k \neq k'$, where K represents the number of states; E_i represents the energy of node i; $P_{k\leftrightarrow k'}$ is a set of paths connecting the local minimum nodes of state k and state k' among neighboring nodes without visiting the same node twice; $\hat{P}_{k\leftrightarrow k'}$ is a subset of $P_{k\leftrightarrow k'}$ that consists of paths passing through nodes belonging to only state k and state k'. For example, a path in $\hat{P}_{1\leftrightarrow 3}$ does not visit nodes in state 2. From these definitions, the element of $P_{k\leftrightarrow k'}$ and $\hat{P}_{k\leftrightarrow k'}$, p_n is a set of nodes belongings to the corresponding paths.

References for Supplementary Text

1. Ezaki T, Watanabe T, Ohzeki M, Masuda N. Energy landscape analysis of neuroimaging data. *Philos Trans A Math Phys Eng Sci*. (2017) 375:20160287. doi: 10.1098/rsta.2016.0287

2 Supplementary Tables

Supplementary Table 1. Background characteristics of the study participants†.

Item name	All	First ELA	Second ELA	Missing (n)
Participants (n)	18,373	4,928	242	-
Diabetes (n)	1,192 (6.5%)	242 (4.9%)	242 (100%)	-
Male (n)	10,454 (57%)	4,928 (100%)	242 (100%)	-
Records (n)	108,920	22,326	766	-
Fasting records (n)	60,369 (55%)	22,326 (100%)	766 (100%)	-
Period (year)	6.33 ± 3.23	5.26 ± 3.21	3.47 ± 2.16	-
Age (years)	55.8 ± 3.3	56.0 ± 3.2	54.5 ± 2.8	0 (0.0%)
BMI (kg/m^2)	23.1 ± 3.5	23.6 ± 3.2	26.1 ± 3.7	3,185 (2.9%)
WC (cm)	83.7 ± 9.6	85.1 ± 8.9	91.5 ± 9.3	4,167 (3.8%)
SBP (mmHg)	126.5 ± 16.5	127.2 ± 14.9	129.5 ± 13.9	2,799 (2.6%)
DBP (mmHg)	77.4 ± 11.8	79.9 ± 10.9	81.5 ± 10.3	2,799 (2.6%)
TG (mg/dL)	131.0 ± 111.3	130.2 ± 105.8	171.7 ± 136.5	4,371 (4.0%)
LDL-C (mg/dL)	128.6 ± 32.1	127.9 ± 31.2	133.1 ± 34.2	4,377 (4.0%)
HDL-C (mg/dL)	63.2 ± 16.4	59.7 ± 15.0	51.9 ± 14.2	4,370 (4.0%)
PG (mg/dL)	99.2 ± 23.8	96.1 ± 10.6	111.9 ± 12.0	7,051 (6.5%)
HbA1c (%)	5.75 ± 0.58	5.65 ± 0.35	6.28 ± 0.42	39,808 (37%)
HbA1c (mmol/mol)	39.3 ± 6.4	38.3 ± 3.8	45.1 ± 4.6	39,808 (37%)
UA (mg/dL)	5.56 ± 1.38	6.17 ± 1.25	6.24 ± 1.37	29,963 (28%)
Cre (mg/dL)	0.79 ± 0.34	0.88 ± 0.38	0.84 ± 0.13	31,099 (29%)
AST (U/L)	24.2 ± 16.7	24.9 ± 15.5	31.6 ± 56.7	4,382 (4.0%)
ALT (U/L)	23.6 ± 18.5	25.8 ± 16.7	39.2 ± 28.6	4,382 (4.0%)
γ-GTP (U/L)	46.2 ± 64.0	54.2 ± 61.2	78.6 ± 81.9	4,382 (4.0%)
WBC (1/μL)	6144 ± 1924	6121 ± 1726	6909 ± 1960	9,059 (8.3%)
RBC (10k/μL)	468.9 ± 42.5	486.5 ± 39.5	501.2 ± 36.7	5,557 (5.1%)
Hb (g/dL)	14.3 ± 1.4	15.1 ± 1.1	15.6 ± 1.0	5,557 (5.1%)
Ht (%)	42.8 ± 3.7	44.8 ± 3.0	46.1 ± 2.8	8,045 (7.4%)

†The meanings of abbreviations are shown in Supplementary Table 2. The missing values were counted for all data, and there were no missing values for the first and second ELA.

Supplementary Table 2. List of Abbreviations

Abbreviation	Full name
ALT	Alanine aminotransferase
AST	Aspartate aminotransferase
BMI	Body mass index
Cre	Creatinine
DBP	Diastolic blood pressure
ELA	Energy landscape analysis
Hb	Hemoglobin
HbA1c	Hemoglobin A1c / Glycated hemoglobin
HDL-C	High-density lipoprotein-cholesterol
Ht	Hematocrit
LDL-C	Low-density lipoprotein-cholesterol
ML	Machine learning
PG	Plasma glucose
RBC	Red blood cell count
ROC	Receiver operating characteristic
SBP	Systolic blood pressure
TG	Triglyceride
UA	Uric acid
WBC	White blood cell count
WC	Waist circumference
γ-GTP	γ-glutamyl transpeptidase

Supplementary Table 3. Comparison of feature values in the year before the transition for the second ELA†.

Feature	state 1 to 3	state 2 to 4	p-value
BMI (kg/m^2)	23.8 ± 2.3	29.3 ± 3.4	≤ 0.0001
WC (cm)	84.9 ± 5.0	99.1 ± 8.9	≤ 0.0001
SBP (mmHg)	126.7 ± 15.4	130.2 ± 11.9	0.13
DBP (mmHg)	78.7 ± 10.1	84.5 ± 9.7	≤ 0.01
TG (mg/dL)	126.7 ± 64.0	181.6 ± 91.4	≤ 0.01
LDL-C (mg/dL)	125.9 ± 29.6	135.1 ± 29.1	0.30
HDL-C (mg/dL)	55.3 ± 13.0	44.6 ± 9.0	≤ 0.001
PG (mg/dL)	112.0 ± 6.6	108.6 ± 7.3	≤ 0.05
HbA1c (%)	6.28 ± 0.31	6.25 ± 0.3	0.70
HbA1c (mmol/mol)	45.1 ± 3.4	44.8 ± 3.3	0.70
UA (mg/dL)	5.82 ± 1.36	7.22 ± 1.22	≤ 0.001
Cre (mg/dL)	0.81 ± 0.12	0.87 ± 0.15	0.13
AST (U/L)	23.9 ± 7.5	37.2 ± 16.0	≤ 0.0001
ALT (U/L)	26.7 ± 15.9	53.0 ± 23.9	≤ 0.0001
γ-GTP (U/L)	62.0 ± 92.5	76.6 ± 60.9	≤ 0.01
WBC (1/μL)	7146 ± 2625	7167 ± 1801	0.58
RBC (10k/μL)	496.1 ± 40.2	511.6 ± 34.1	0.16
Hb (g/dL)	15.5 ± 1.1	15.9 ± 1.0	0.22
Ht (%)	45.8 ± 3.1	46.7 ± 2.6	0.24

†The meanings of abbreviations are shown in Supplementary Table 2.

3 Supplementary Figures

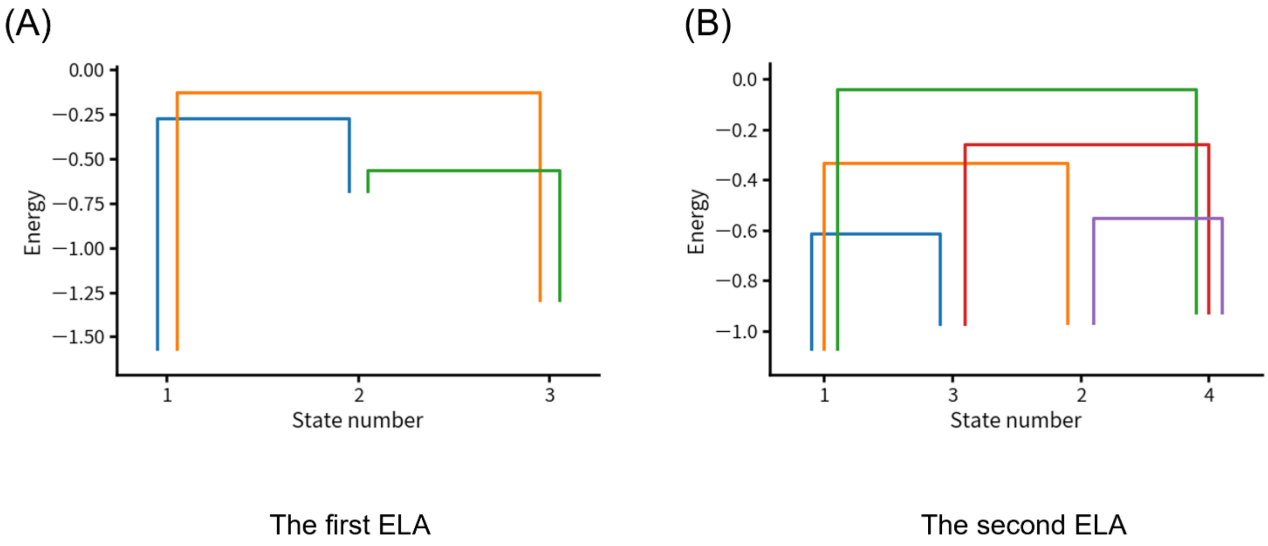

Supplementary Figure 1. Modified disconnectivity graphs. (A) The first ELA. (B) The second ELA. Note that the first and second ELA states have different meanings. See the main text for details. ELA: energy landscape analysis.

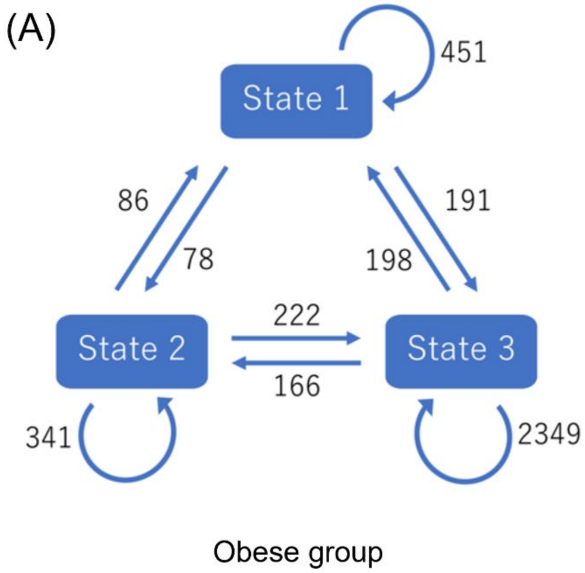

Supplementary Figure 2. State transition counts for obese and non-obese groups for the first energy landscape analysis. (A) Obese group with BMI ≥ 25 kg/m^2 (n=1,460). (B) Non-obese group with BMI < 25 kg/m^2 (n=3,468). BMI: body mass index.
